# Modeling Ca^2+^-Bound Troponin in Excitation Contraction Coupling

**DOI:** 10.3389/fphys.2016.00406

**Published:** 2016-09-21

**Authors:** Henry G. Zot, Javier E. Hasbun

**Affiliations:** ^1^Department of Biology, University of West GeorgiaCarrollton, GA, USA; ^2^Department of Physics, University of West GeorgiaCarrollton, GA, USA

**Keywords:** contraction, calcium, troponin, excitation, muscle, EC-coupling, model, kinetics

## Abstract

To explain disparate decay rates of cytosolic Ca^2+^ and structural changes in the thin filaments during a twitch, we model the time course of Ca^2+^-bound troponin (Tn) resulting from the free Ca^2+^ transient of fast skeletal muscle. In fibers stretched beyond overlap, the decay of Ca^2+^ as measured by a change in fluo-3 fluorescence is significantly slower than the intensity decay of the meridional 1/38.5 nm^−1^ reflection of Tn; this is not simply explained by considering only the Ca^2+^ binding properties of Tn alone (Matsuo et al., 2010). We apply a comprehensive model that includes the known Ca^2+^ binding properties of Tn in the context of the thin filament with and without cycling crossbridges. Calculations based on the model predict that the transient of Ca^2+^-bound Tn correlates with either the fluo-3 time course in muscle with overlapping thin and thick filaments or the intensity of the meridional 1/38.5 nm^−1^ reflection in overstretched muscle. Hence, cycling crossbridges delay the dissociation of Ca^2+^ from Tn. Correlation with the fluo-3 fluorescence change is not causal given that the transient of Ca^2+^-bound Tn depends on sarcomere length, whereas the fluo-3 fluorescence change does not. Transient positions of tropomyosin calculated from the time course of Ca^2+^-bound Tn are in reasonable agreement with the transient of measured perturbations of the Tn repeat in overlap and non-overlap muscle preparations.

## Introduction

During a twitch of striated muscle, the intracellular fluorescence probe fluo-3 reveals two types of calcium transients (Minta et al., 1989). Owing to its high affinity for Ca^2+^, fluo-3 detects and contributes to a cytosolic pool of Ca^2+^ that rises rapidly and persists longer than 150 ms after stimulation of frog striated muscle at 16°C (Harkins et al., 1993; Caputo et al., 1994; Matsuo et al., 2010). The long decay time can be explained by Ca^2+^ exchange with binding molecules in the cytosol, which may be immobilized molecules such as troponin (Tn) or diffusive molecules such as ATP and parvalbumin, during sequestration of Ca^2+^ by the sarcoplasmic reticulum (Baylor and Hollingworth, 2011). A transient of free Ca^2+^, well described by the low-affinity probe furaptra (Hollingworth et al., 2009), can also be calculated from the fluo-3 record (Caputo et al., 1994). The transient of free Ca^2+^ rises to a peak in 5–7 ms and decays to baseline in about 50 ms at 16°C (Konishi et al., 1991; Hollingworth and Baylor, 2013). This brief pulse of Ca^2+^ produced by Ca^2+^ sparks (Cannell et al., 1995) constitutes the intracellular excitation signal for myofilament contraction (Baylor et al., 2002; Baylor and Hollingworth, 2007).

The Ca^2+^ regulatory sites of Tn (Potter and Gergely, 1975) mediate excitation contraction coupling (Robertson et al., 1981). Based on the binding characteristics of purified Tn (Baylor and Hollingworth, 1998), the decay of Ca^2+^-bound Tn is expected to follow the long process of fluo-3 decay (Matsuo and Yagi, 2008). However, a simple Ca^2+^ dissociation rate of Tn is difficult to reconcile with the modeling of the furaptra transient (Baylor et al., 2002). In muscle preparations stretched to average sarcomere lengths of 2.8 and 4.0 μm (overlap and non-overlap preparations, respectively), decays of fluo-3 signals are remarkably similar, but, in the non-overlap preparation, the intensity of the meridional 1/38.5 nm^−1^ reflection corresponding to the repeat of Tn in the thin filament decays significantly faster than the fluo-3 signal (Matsuo and Yagi, 2008). If Ca^2+^-bound Tn is a function of the pool of Ca^2+^ represented by the fluo-3 signal then a significant fraction of Tn remains in the Ca^2+^-bound state after the Tn-related structure fully relaxes (Matsuo and Yagi, 2008). Our aim is to provide a theoretical framework for the alternative hypothesis, namely, the structure represented by the meridional 1/38.5 nm^−1^ reflection has a direct relationship with Ca^2+^-bound Tn.

The properties of Ca^2+^ binding to the regulatory sites of the C-subunit of Tn (TnC) depend on interactions of Tn with actin in a 7:1:1 molar complex of actin, tropomyosin (Tm), and Tn (regulated actin). Studies using both ^45^Ca^2+^ and fluorescence change techniques with native and covalently modified preparations, respectively, consistently demonstrate that the Ca^2+^ affinity of regulated actin is substantially lower than the Ca^2+^ affinity of isolated Tn (Wnuk et al., 1984; Rosenfeld and Taylor, 1987; Zot, H. G. and Potter, J. D., 1987). Kinetic measurements of Ca^2+^-dependent fluorescence changes show slow and fast rates of Ca^2+^ dissociation from regulated actin; the slow rate correlates with the Ca^2+^ dissociation rate of isolated Tn, while the other rate is about 10-fold faster (Rosenfeld and Taylor, 1987). Rigor myosin shifts the affinity of regulated actin (myosin:actin:Tm:Tn in a 7:7:1:1 complex with no ATP) to the higher Ca^2+^ affinity of isolated Tn and reduces the kinetic measurement to one rate, which matches the slow rate of Ca^2+^ dissociation (Rosenfeld and Taylor, 1987). Tropomyosin can occupy three different positions relative to actin: blocking (*B*), central (*C*), and myosin dependent (*M*) positions. Tn in association with Tm can interact with actin only when Tm is in position *B* (Lehman et al., 2000). A competition between the open conformation of TnC and actin for the same internal structure of Tn in position *B* (Gagné et al., 1995; Takeda et al., 2003) could lower the apparent Ca^2+^ affinity and increase the Ca^2+^ off rate of Tn in position *B* by energy coupling. By the same energetic principle, when Tm is in either position *C* or *M* and Tn cannot interact with actin, the regulatory sites of TnC should have the higher Ca^2+^ affinity and slower Ca^2+^ off rate of isolated Tn.

Cooperative changes associated with Ca^2+^ binding to TnC depend on not only the context of regulated actin but also the context of rigor and steady-state conditions. Although, some preparations of fluorescently modified TnC display cooperative Ca^2+^-dependent fluorescence changes (Grabarek et al., 1983; Zot, H. G. and Potter, J. D., 1987; Davis et al., 2002), only a single class of non-interacting Ca^2+^-binding sites is found for the regulatory sites of native and fluorescently modified TnC in regulated actin by techniques using ^45^Ca^2+^ and fluorescence change, respectively (Wnuk et al., 1984; Rosenfeld and Taylor, 1987; Zot, H. G. and Potter, J. D., 1987). Likewise, a non-cooperative fluorescence change in response to Ca^2+^ is observed for regulated actin saturated with rigor myosin (Rosenfeld and Taylor, 1987). However, in the presence of ATP, muscle fibers and myofibrils reconstituted with fluorescent TnC display steeply cooperative Ca^2+^-dependent activation and fluorescence changes (Zot et al., 1986; Zot, A. S. and Potter, J. D., 1987; Brandt and Poggesi, 2014). Hence, cooperative Ca^2+^ binding requires steady-state crossbridges.

Here we link the well-described transient of free Ca^2+^ to a comprehensive model of contraction (Zot et al., 2009). This model accounts for Ca^2+^-bound Tn in association with Tm in the three principle structural states of the thin filament (Lehman et al., 2000). As with regulated actin, the muscle fiber is expected to display both slow and fast Ca^2+^ dissociation rates, which should be evident in the decay rates of structural changes related to Tn and also depend on cycling crossbridges. We apply the model to transient changes in the fluo-3 fluorescence and meridional 1/38.5 nm^−1^ reflection intensities measured in preparations of frog skeletal muscle at 16°C, with the sarcomere length maintained at overlap or non-overlap of myofilaments (Matsuo et al., 2010), which promotes or prohibits cycling crossbridges, respectively. The model presented here predicts that Ca^2+^-bound Tn follows the slow decays of fluo-3 fluorescence and meridional 1/38.5 nm^−1^ reflection intensities of the overlap preparation and only the faster decay of meridional 1/38.5 nm^−1^ reflection intensity of the non-overlap preparation. The pool of Ca^2+^ represented by the fluo-3 fluorescence intensity and Ca^2+^-bound Tn lack a predictable relationship.

## Materials and methods

### Description of model

**Figure 1:**
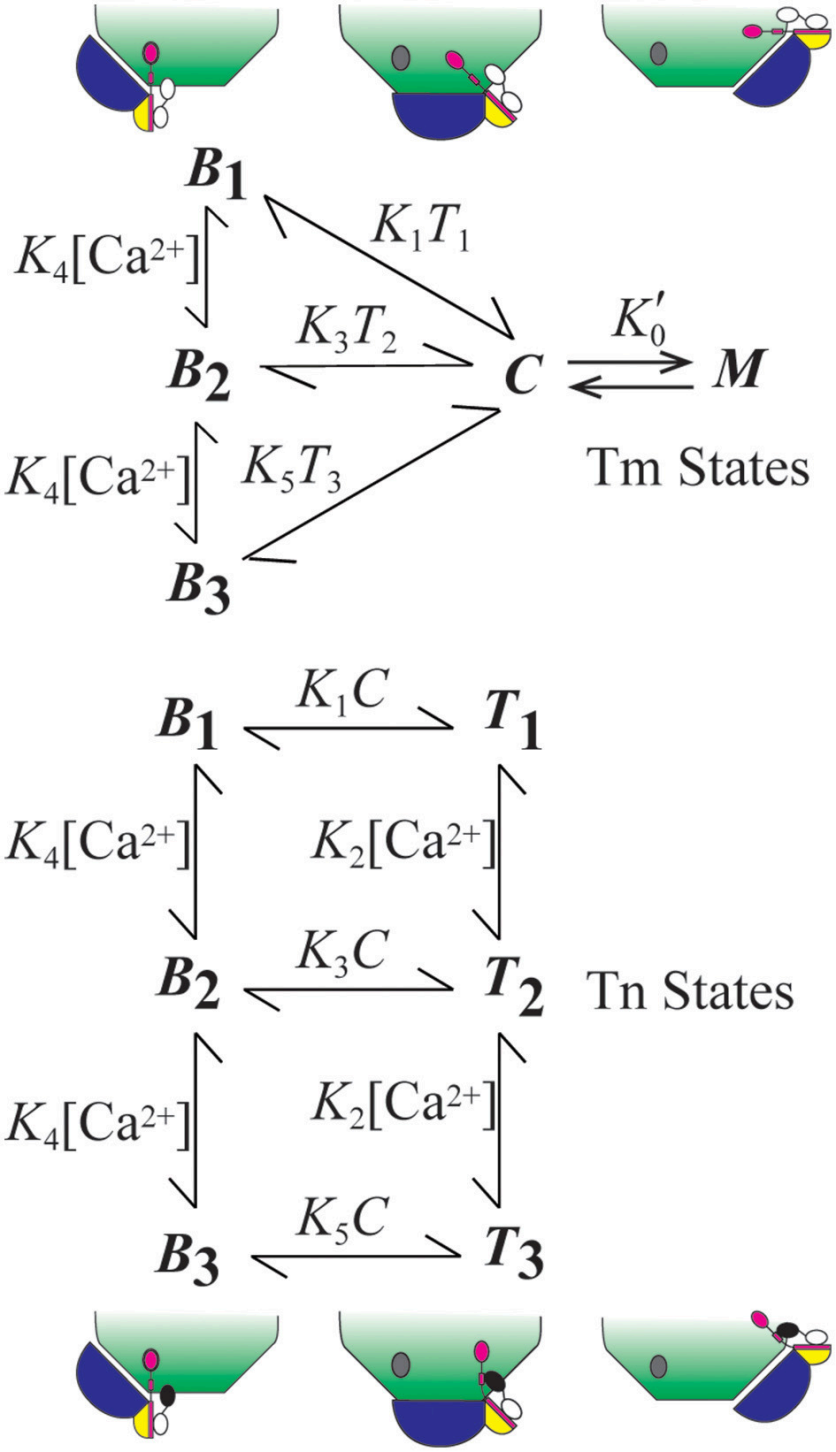
**Summary of model**. The model consists of two subsystems that partially overlap. The states of Tm (blue) include central (*C*), myosin-dependent (*M*), and blocking (*B*_*i*_, *i* = 1–3). States of Tn (TnT, yellow; TnI, magenta; TnC, black-white) include energetically coupled (*B*_*i*_) and uncoupled (*T*_*i*_) states each having regulatory sites of TnC that can be Ca^2+^-free (*i* = 1; white), singly Ca^2+^-bound (*i* = 2; black), and doubly Ca^2+^-bound (*i* = 3; black). The diagram shows how Tn is isolated from a site of interaction with actin (gray oval) by the movement of Tm from position *B* to positions *C* and *M*. All constants depicted in the figure represent the ratio of component rate constants (Table 1) used to calculate the relative abundance or probability of each state as a function of a transient change in calcium concentration.

The model we employ accounts for the relative distributions of thin filament states (Figure 1). The *B, C*, and *M* states of Tm refer to Tm's interactions with actin in these respective positions (Lehman et al., 2000). State *C* is the equilibrium position (Phillips et al., 1986; Lehman et al., 2000), and states *B* and *M* are modeled as competing for Tm in state *C*. To stabilize the non-equilibrium positions *B* and *M*, Tm-bound Tn forms an interaction with actin (Greaser and Gergely, 1973) in position *B*, and Tm forms a ternary complex with crossbridges and actin in position *M* (Eaton, 1976; Tobacman and Butters, 2000). The interaction of Tn in state *B* accounts for the states of Tn that are energetically coupled to the states of Tm. Movement of Tm away from *B* energetically uncouples Tn from possible interactions with actin (Figure 1).

Coupled and uncoupled states of Tn are designated *B* and *T*, respectively (Figure 1). Calcium-dependent states of Tn are designated *B*_*i*_ and *T*_*i*_, where *i* represents 1 (Ca^2+^ free), 2 (singly bound), or 3 (doubly bound). Affinity for actin is progressively reduced as *i* increases. Based on conservation of the mass for two partially overlapping subsystems (Tm and Tn), *B* + *C* + *M* = 1 for Tm and *B* + *T* = 1 for Tn.

In the system we describe, *M* can arise by either rigor or cycling crossbridges, but cooperativity derives solely from cycling crossbridges, as seen in the records of Ca^2+^ binding measurements. With overlapping thin and thick filaments and ATP, *M* is a state in constant flux (steady-state) rather than at equilibrium; this is readily observed by Ca^2+^-dependent *in vitro* sliding of regulated actin filaments. Steady-state cooperativity is achieved if crossbridge turn-over generates additional opportunities (second chances) for *M* formation (Zot et al., 2009, 2012). By analogy, the *M* state operates like a man whose feet are bound to a ceiling by adhesion: changing positions quickly relative to the rate of deadhesion improves the odds of remaining bound by re-establishing the initial binding conditions. A statistical treatment of a second chance mechanism applied to data from biological systems is available (Zot et al., 2016a). In practice, a second chance mechanism is given in the following rate equation

(1) $$dM/dt=K_{0}^{\prime }k_{-0}C{\left(1+(\alpha -1)M\right)}^{n}-k_{-0}M$$

where parameters $K_{0}^{\prime }$, *k*_−0_, α, and *n* are derived elsewhere (Zot et al., 2009, 2012). The parameter α expresses second chance opportunities for reestablishing equilibrium before the decay of *M*. As steady-state or equilibrium approach, *dM*/*dt* → 0. Because Equation (1) acts on the *C*-*M* transition (Figure 1), cooperativity does not directly involve Ca^2+^ binding to Tn. Although, Equation (1) performs adequately, any logistic function operating on *C* can be compatible with our model.

As applied to transient and steady-state striated muscle regulation, the parameters of Equation (1) may have the following interpretations. The equilibrium potential of *M* as a function of the population of strong binding myosin at any moment of steady-state is expressed by $K_{0}^{\prime }$. The forward rate of ensemble *M* formation is the product $K_{0}^{\prime }$*k*_−0_. Ensemble size, *n*, expresses the number of Tm subunits acting in concert to form a ternary complex as described above. The orchestrating event could be a lateral stretch imposed on contiguous Tm subunits by an axial force acting on the thin filament (Zot et al., 2009). The parameter α is an expression of the crossbridges poised to replace crossbridges disrupted by internal chemomechanical forces or by active sliding. The value of α may be related to the average number of crossbridges in a target zone (Tregear et al., 2004), as has been described (Zot et al., 2009). We give unit value to $K_{0}^{\prime }$ for simplicity and recycle previously discussed values for α, and *n* (Zot et al., 2009; Table 1) for consistency.

**Table 1:** **Summary of standard conditions**.

**Steady-state constant**		**Component rate constant**		
	**Value used^a^**		**Value used**	**Dimensions**
$K_{0}^{\prime }$	1	$K_{0}^{\prime }$ *k*_−0_	50	s^−1^
		*k*_−0_	50	s^−1^
*K*_1_	800	*K*_1_ *k*_−1_	80,000	s^−1^
		*k*_−1_	100	s^−1^
*K*_3_	80	*K*_3_ *k*_−3_	8000	s^−1^
		*k*_−3_	100	s^−1^
*K*_5_	8	*K*_5_ *k*_−5_	800	s^−1^
		*k*_−5_	100	s^−1^
*K*_2_	1.67	*K*_2_ *k*_−2_	25	μM^−1^s^−1^
		*k*_−2_	15	s^−1^
*K*_4_	0.167	*K*_4_ *k*_−4_	25	μM^−1^s^−1^
		*k*_−4_	150	s^−1^
*n*	3.25			
α	5			

a *Shown previously to fit steady-state Ca^2+^-dependent tension of skinned fibers of fast twitch (Zot et al., 2009) and slow twitch (Zot et al., 2016b) muscle*.

### Computational methods

Transitions other than *C-M* are spontaneous processes governed by simple mass action (Figure 1). Although the model has eight states, only six are independent. If we choose to calculate states *C* and *T*_1_ by mass conservation (see above), the relative abundances or probabilities of the other six states (Figure 1) as a function of an independent calcium transient are calculated by solving a system of six ordinary differential equations (ODE), i.e., in addition to Equation (1), we have

$$\begin{array}{l} dB_{1}/dt=K_{1}k_{-1}CT_{1}+k_{-4}B_{2}-\left(k_{-1}+K_{4}k_{-4}[Ca^{2+}]\right)B_{1};\\ dB_{2}/dt=K_{3}k_{-3}CT_{2}+k_{-4}B_{3}+K_{4}k_{-4}[Ca^{2+}]B_{1}\\ \;-\left(k_{-3}+k_{-4}+K_{4}k_{-4}[Ca^{2+}]\right)B_{2};\\ dB_{3}/dt=K_{5}k_{-5}CT_{3}+K_{4}k_{-4}[Ca^{2+}]B_{2}-\left(k_{-4}+k_{-5}\right)B_{3};\\ dT_{2}/dt=K_{2}k_{-2}[Ca^{2+}]T_{1}+k_{-3}B_{2}+k_{-2}T_{3}\\ \;-\left(k_{-2}+K_{2}k_{-2}[Ca^{2+}]+K_{3}k_{-3}C\right)T_{2};\\ dT_{3}/dt=K_{2}k_{-2}[Ca^{2+}]T_{2}+k_{-5}B_{3}-\left(k_{-2}+K_{5}k_{-5}C\right)T_{3}. \end{array}$$

**Figure 2:**
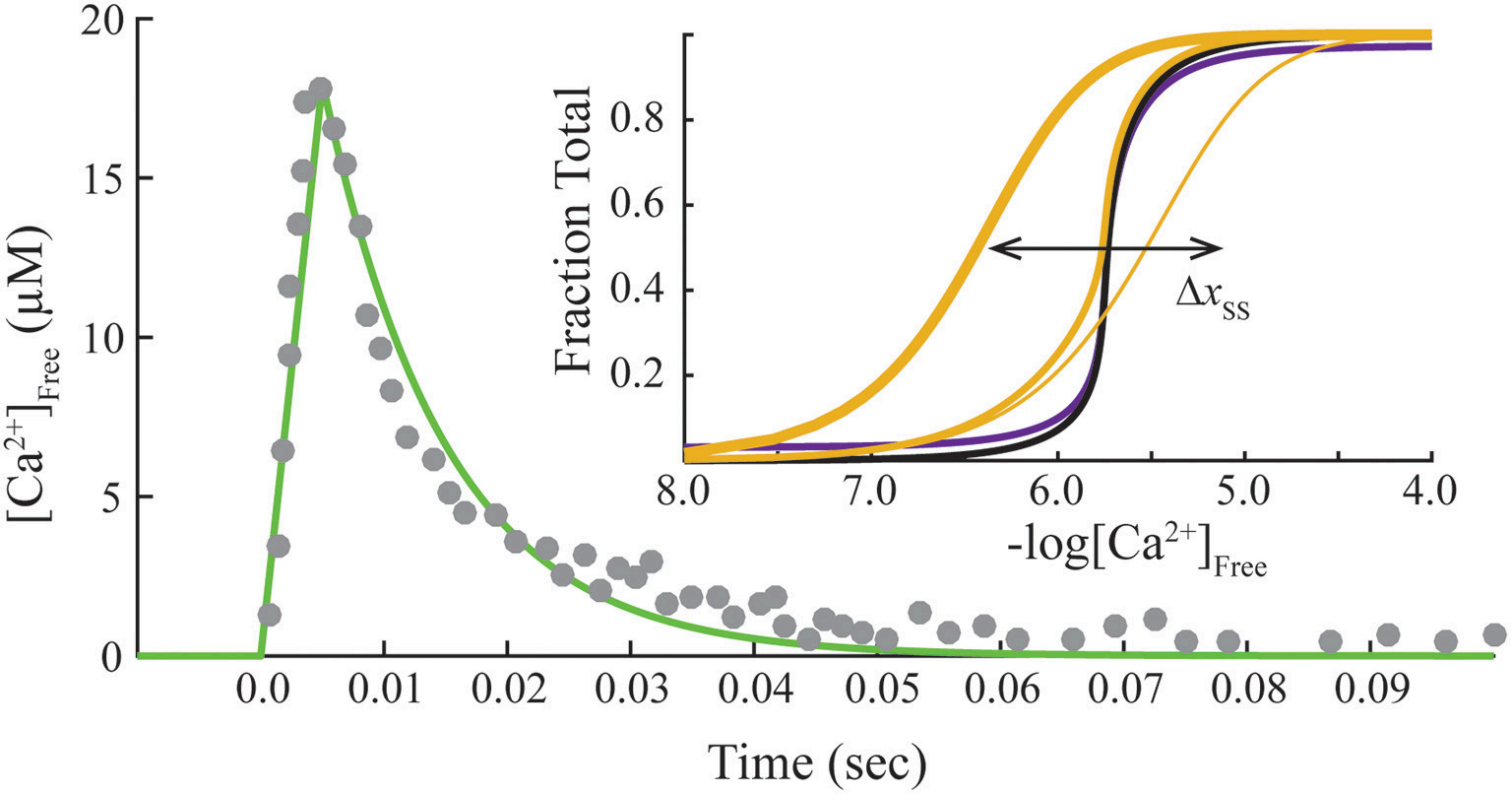
**Standard transient of free Ca^2+^ and final steady-state conditions**. Transient free Ca^2+^ data (dots) reproduced from Matsuo et al. (2010) are fit empirically by a mathematical function (green). Inset, Steady-state activation (*M* state) is plotted as a function of free Ca^2+^ concentration on absolute (purple) and normalized (black) scales. Adjusting steady-state constants (Table 2) shifts the curves for activation laterally (Δ*x*_ss_). Predicted Ca^2+^-bound Tn curves (gold) that simulate no crossbridges (1 pt. line), saturating rigor crossbridges (3 pt. line), and cycling crossbridges (2 pt. gold line) are calculated with $K_{0}^{\prime }$ set to 0, 10^6^, and 1, respectively, under the standard equilibrium/steady-state conditions of Table 1. Hill coefficients (Hill, 1910) of unity are determined for curves representing no crossbridges and rigor crossbridges.

This system of ODE is solved for each free Ca^2+^ concentration of a given transient. The free Ca^2+^ transient of a muscle fiber (Matsuo et al., 2010) is reproduced by a linear rise from the origin to the peak (time to peak is 0.005 s), followed by an exponential decay (rate constant is 100 s^−1^; Figure 2). We use the same Ca^2+^ transient for all calculations as a control. Hence, the model varies only the contribution of crossbridges in fitting data from overlap and non-overlap preparations. A Matlab program is provided to reproduce calculations presented here (see Supplementary Materials).

Standard conditions refer to a set of constants governing steady-state potentials (upper case, “*K*”), α, and *n* that we hold constant (Table 1). Thermodynamic principles dictate that *K*_1_/*K*_3_ = *K*_3_/*K*_5_ = *K*_2_/*K*_4_, which allows $K_{0}^{\prime }$, *K*_1_, *K*_2_, and *K*_4_ to be selected independently. The values used here for these four parameters, α, and *n* (Table 1) are the same shown elsewhere to fit steady-state activation of skinned fibers of fast muscle (Zot et al., 2009) and cardiac muscle (Zot et al., 2016b) by Ca^2+^. Component rate constants (lower case, “*k*”) are varied to fit transient data. Slow and fast dissociation rates of Ca^2+^ from Tn (*k*_−2_ and *k*_−4_; Table 1), are taken from Rosenfeld and Taylor (1987).

## Results

### Steady-state and equilibrium behavior of the model

Steady-state or equilibrium calculations are produced for standard conditions (Table 1) by nullifying the system of ODE (cf. Matlab program in Zot et al., 2016b). Calculated Ca^2+^-dependent activation curves on absolute and normalized scales (inset, Figure 2) reproduce fits of steady-state tension and ATPase data of diverse native and mutant protein preparations of fast skeletal and cardiac muscles (Zot et al., 2009, 2016b). To verify that the model reproduces established Ca^2+^ binding properties of Tn, Ca^2+^-bound Tn (*B*_2_ + *B*_3_ + *T*_2_ + *T*_3_) is calculated as a function of constant concentrations of Ca^2+^. With cycling crossbridges, the mathematical solution is a cooperative function of Ca^2+^ (inset, Figure 2), which reproduces the distinctive binding-activation relationship observed previously with fluorescently labeled Tn in reconstituted myofibrils (Zot et al., 1986; Zot, A. S. and Potter, J. D., 1987; Brandt and Poggesi, 2014). To model equilibrium achieved by non-overlap or rigor, $K_{0}^{\prime }$ is set either to zero or to a relatively large value (10^6^), respectively. Both solutions predict simple mass action (non-cooperative) calcium binding (inset, Figure 2), and both simulations reproduce published simple mass action Ca^2+^ binding curves for regulated actin and regulated actin with rigor myosin binding, respectively (Rosenfeld and Taylor, 1987). Hence, the model is able to reproduce cooperative and non-cooperative Ca^2+^ binding measurements of both steady-state and equilibrium preparations, respectively.

### Transient response with overlap

**Figure 3:**
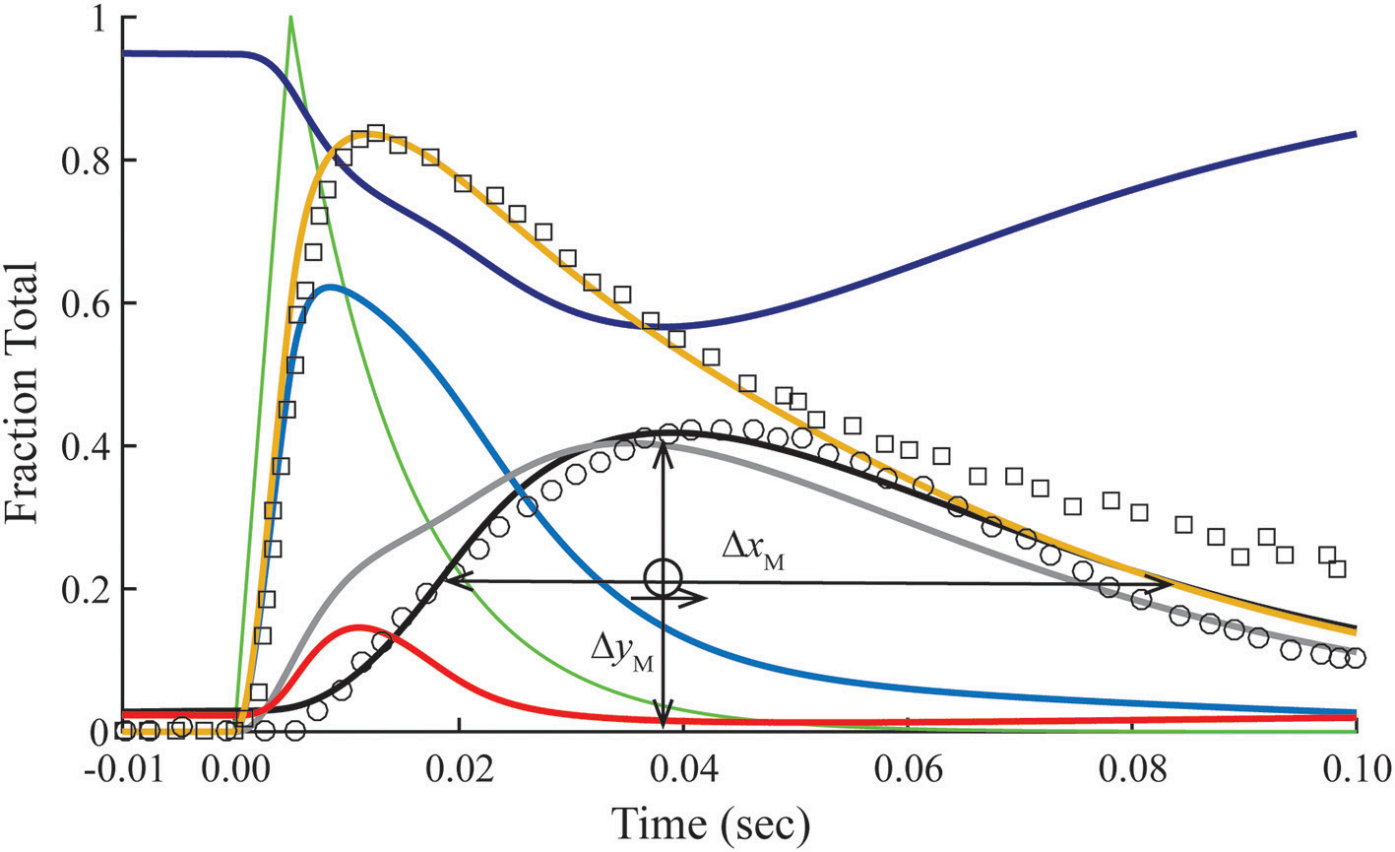
**Time courses of principle states of the thin filament with overlap**. Plotted as a function of a pulse of free calcium (green) are calculated transients of states *B* (dark blue), *C* (red), and *M* (black). Total Ca^2+^-bound Tn (*B*_2_ + *B*_3_ + *T*_2_ + *T*_3_; gold) is broken down into components, i.e., fast (*B*_2_ + *B*_3_; blue) and slow (*T*_2_ + *T*_3_; gray). Plotted on the same scale are measured tension (circles) and fluo-3 fluorescence (squares) transients of muscle fibers of average sarcomere length of 2.8 μm and stimulated by a single impulse (reproduced from Matsuo et al., 2010). Adjustments in standard conditions (Table 1) alter the width (Δ*x*_M_), height (Δ*y*_M_), and center of the *M* peak (O) as described in Table 2.

The transient of calcium-bound troponin, which is modeled as the sum of *B*_2_, *B*_3_, *T*_2_, and *T*_3_, is compared with the transient change measured by fluo-3 fluorescence (Matsuo et al., 2010) in overlap muscle preparations. Rather than choosing parameters to fit the fluo-3 data, we fit tension data (Figure 3) from the same preparation with calculated value of *M* (Equation 1) by adjusting the rate constants (Table 2) that comprise standard conditions (Table 1). Decreasing $K_{0}^{\prime }$*k*_−0_ shifts the calculated tension transient rightward, and *k*_−0_ is decreased in tandem to maintain constant $K_{0}^{\prime }$. The same constraint is used throughout to maintain standard conditions. Although adjusting either $K_{0}^{\prime }$*k*_−0_ or *K*_1_*k*_−1_ changes the tension transient equivalently, using $K_{0}^{\prime }$*k*_−0_ for lateral adjustments and *K*_1_*k*_−1_ for vertical adjustments yields the best shape of the tension transient relative to the data. Given the optimum fit of the tension data, the predicted Ca^2+^-bound Tn transient is seen to fit most of the fluo-3 fluorescent data (Figure 3). If we accept a slightly faster time to peak tension, the model predicts slower decay of Ca^2+^-bound Tn, which may better capture the entire trend of fluo-3 data (see Supplementary Materials).

**Table 2:** **Response to adjustments of standard conditions**.

**Variable**	**Figure**	**Variable response**	**Parameter adjustment**
Δ*x*_SS_	2	Lateral shift	*K*_0_/*K*_1_
Δ*x*_M_	3	Increase peak width	Decrease $K_{0}^{\prime }$ *k*_−0_
			Decrease *K*_1_ *k*_−1_
Δ*y*_M_	3	Increase peak height	Increase $K_{0}^{\prime }$ *k*_−0_
			Increase *K*_1_ *k*_−1_
O	3	Right lateral shift	Decrease $K_{0}^{\prime }$ *k*_−0_
			Decrease *K*_1_ *k*_−1_
Δ*x*_CaB_	5	Leftward shift	Increase *K*_4_ *k*_−4_
Δ*dif*_C_	5	Left relative shift	Increase *K*_1_ *k*_−1_
Δ*x*_CaB_	6	Leftward shift	Increase *K*_1_ *k*_−1_

The dissociation of Ca^2+^ from the Ca^2+^ regulatory sites of Tn is not uniform over time. Owing to a faster off-rate, Ca^2+^-bound *B* states (*B*_2_ + *B*_3_) release Ca^2+^ faster than Ca^2+^-bound *T* states (*T*_2_ + *T*_3_; Figure 3). Hence, a significant fraction of Tn has released Ca^2+^ before tension reaches a peak. A protracted dissociation of residual Ca^2+^ comes mainly from *T* states, which represent a pool of Tn molecules held away from interaction with actin in the *B* position owing to crossbridges (crossbridge-dependent Ca^2+^-bound Tn).

### Transient response with non-overlap

**Figure 4:**
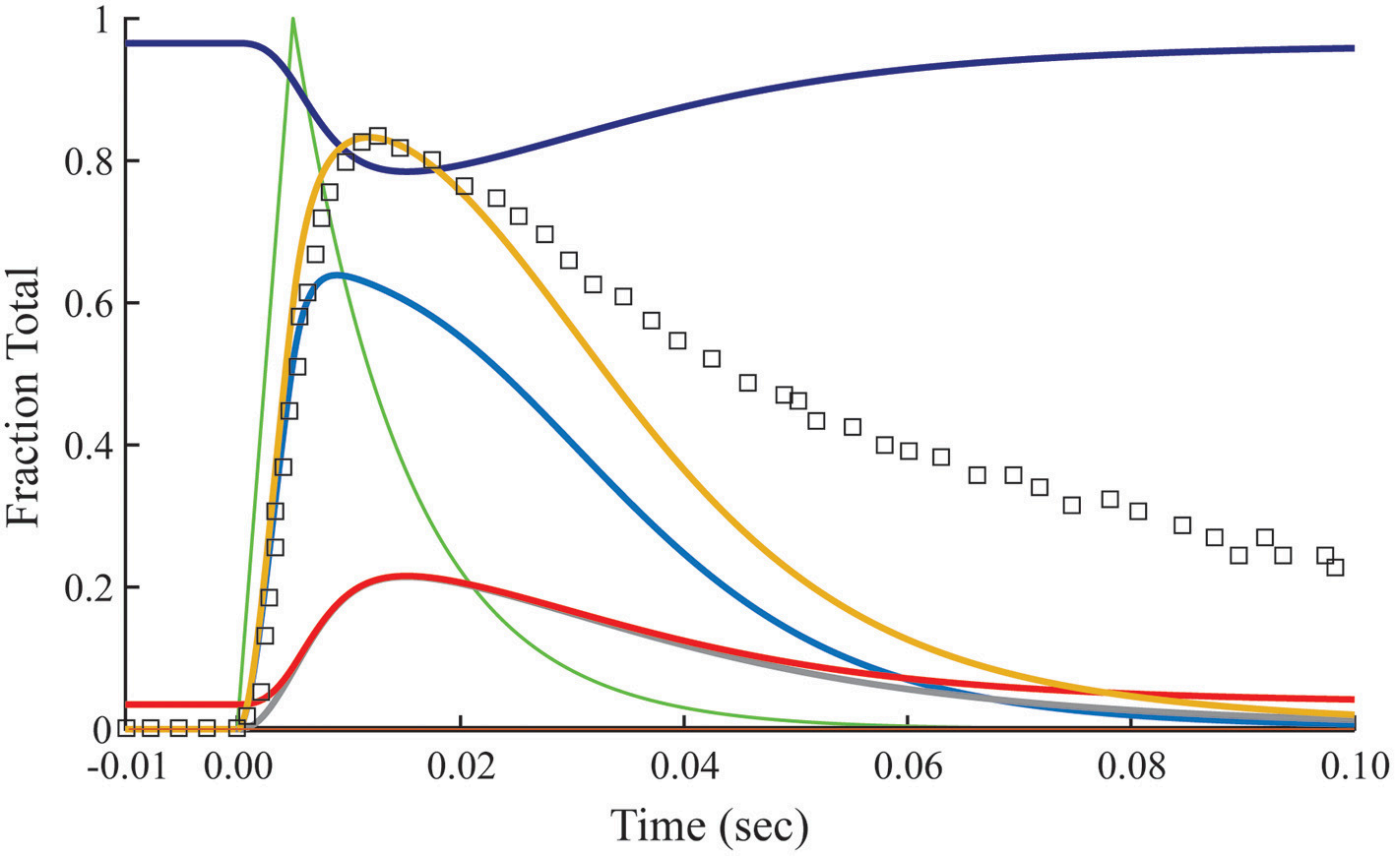
**Time courses of principle states of the thin filament with no overlap**. Plotted as a function of a pulse of free calcium (green) are calculated transients of states *B* (dark blue), *C* (red), and *M* (black). Total Ca^2+^-bound Tn (*B*_2_ + *B*_3_ + *T*_2_ + *T*_3_; gold) is broken down into fast (*B*_2_ + *B*_3_; blue) and slow (*T*_2_ + *T*_3_; gray) components. For comparison, the measured fluo-3 fluorescence transient (squares) is reproduced from Figure 3.

**Figure 5:**
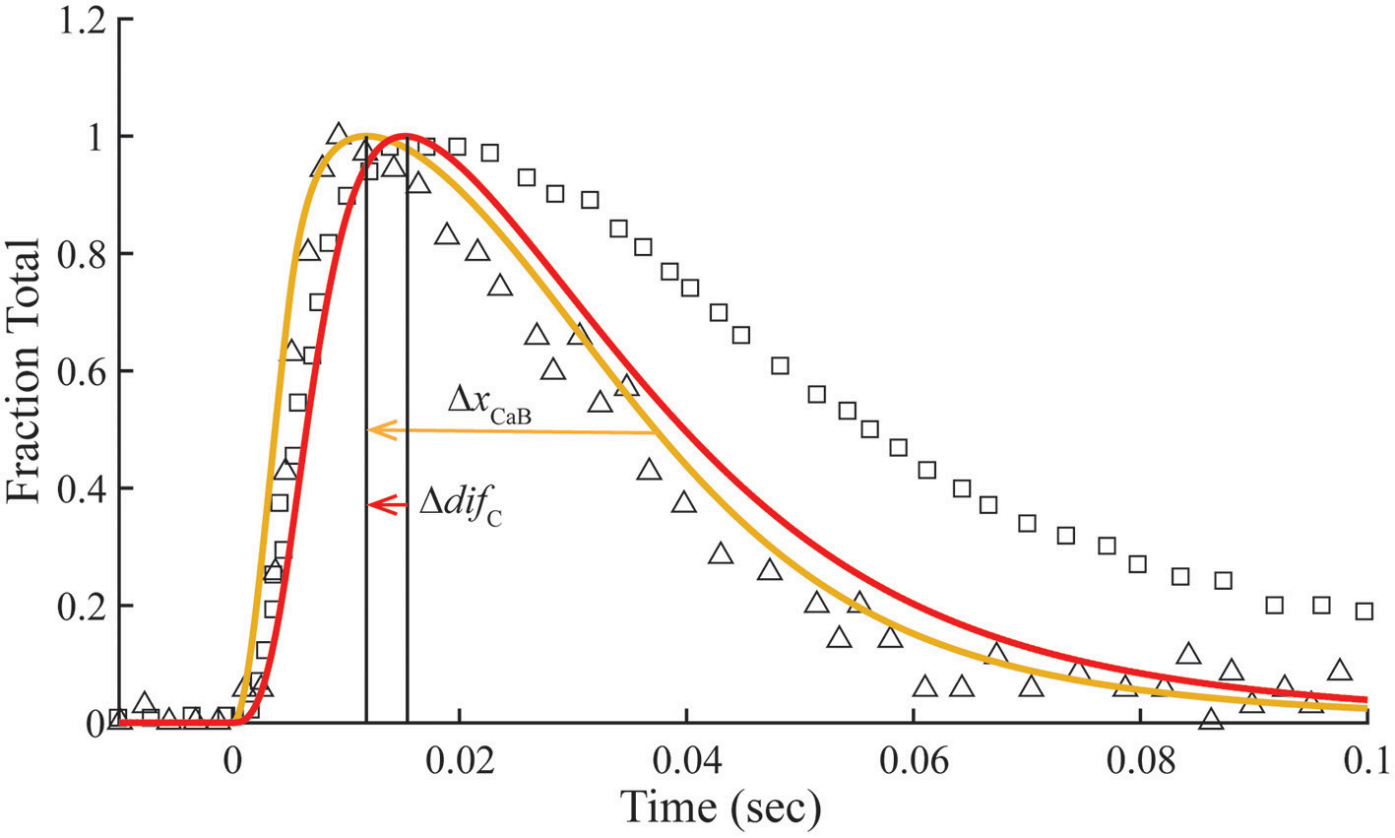
**Time courses of fluo-3 fluorescence and transient structural changes of troponin in nonoverlap sarcomeres**. Changes in intensity of the meridional 1/38.5 nm^−1^ reflection (triangles) and of fluo-3 fluorescence (squares) in response to a single calcium pulse are reproduced for muscle stretched beyond overlap (Matsuo et al., 2010). Plotted on the same scale in response to a simulated calcium pulse (Figure 2) are the calculated temporal change of state *C* (red) and calcium bound to troponin (gold) under standard conditions (Table 1) with $K_{0}^{\prime }$ set to null. Other adjustments in standard conditions (Table 2) alter the decay rate of Ca^2+^-bound Tn (Δ*x*_CaB_) and the relationship with the state *C* transient (Δ*dif*_C_).

The relationship between calculated Ca^2+^-bound Tn and fluo-3 data differs dramatically in muscle fibers stretched beyond overlap. To simulate no overlap, we nullify $K_{0}^{\prime }$*k*_−0_ in Equation (1), but otherwise preserve the rates established for the overlap condition (Table 1). Absent crossbridges, the decays of calculated Ca^2+^-bound troponin, whether expressed as a total or separated into components *B* and *T*, are much faster than the decay of fluo-3 fluorescence (Figure 4). No combinations of rate constants will allow the model to fit the tension data of the overlap preparation and the fluo-3 data of overlap and non-overlap preparations. By contrast, the decay of fluo-3 fluorescence is constant for overlap and non-overlap conditions (Figures 3, 5). Therefore, there is not a causal relationship between the calculated Ca^2+^-bound state of troponin and fluo-3 fluorescence.

Matsuo et al. (2010) show that in a non-overlap sarcomere preparation, the fluo-3 fluorescence decay is slower than the decay of meridional 1/38.5 nm^−1^ reflection intensity, which corresponds to the troponin repeat in the thin filament (Figure 5). We find that the calculated time courses of Ca^2+^-bound Tn and the *C* position of Tm correlate with the transient of meridional 1/38.5 nm^−1^ reflection intensity (Figure 5). A systematic sensitivity test of all rate constants shows that the decay of Ca^2+^-bound Tn is limited only by *k*_−4_ of the model (Figure 5, Table 2). This is the faster of two dissociation rates determined for Ca^2+^ from regulated actin (Rosenfeld and Taylor, 1987). Increasing *k*_−4_ by a factor of 1.33, which is within the range of measured values (Rosenfeld and Taylor, 1987), and maintaining the same steady-state conditions (inset, Figure 2) bring the decay rate of Ca^2+^-bound Tn closer in alignment with the decay of meridional 1/38.5 nm^−1^ reflection intensity (see Supplementary Materials). Furthermore, the decays of Ca^2+^-bound Tn and state *C* of Tm align more closely by increasing *K*_1_*k*_−1_ (Figure 5, Table 2; see Supplementary Materials). Hence, the model predicts that the decay rate of meridional 1/38.5 nm^−1^ reflection intensity in the non-overlap preparation gives an *in vivo* measure of the rate of Ca^2+^ dissociation from Tn. A characteristic intensity increase in response to Ca^2+^ represents Tn-dependent structural changes of the thin filament (Yagi, 2003). Modeling suggests that Ca^2+^-bound Tn regulates completely the structural changes related to the movement of Tm to position C in the non-overlap preparation.

### Transient structural changes with overlap

**Figure 6:**
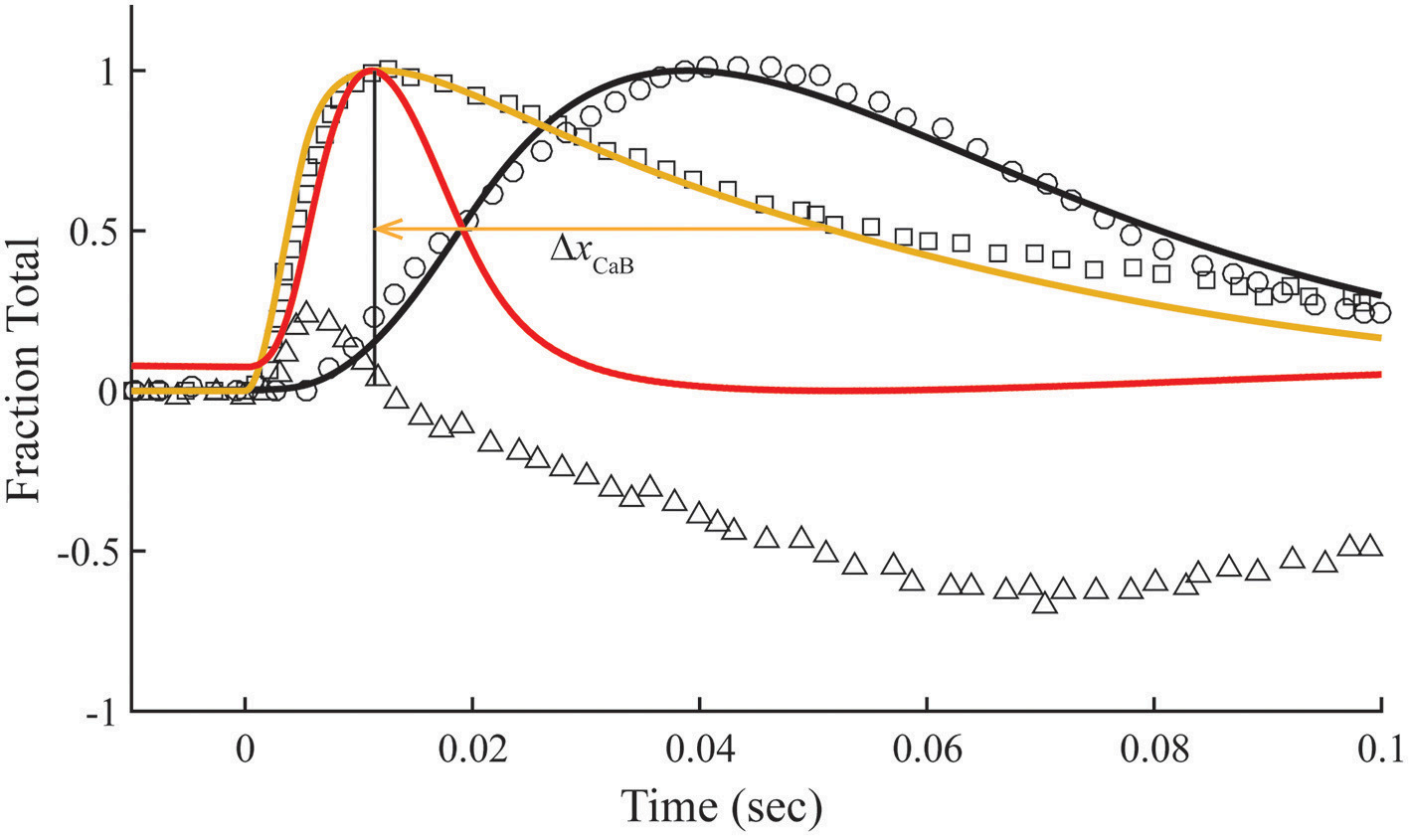
**Model compared with temporal responses measured in overlap sarcomeres**. Intensities of the meridional 1/38.5 nm^−1^ reflection (triangles), tension (circles), and fluo-3 fluorescence (squares) for average sarcomere length of 2.8 μm in response to a single calcium pulse are reproduced (Matsuo et al., 2010). Plotted on the same scale in response to a simulated calcium pulse (Figure 2) are the calculated temporal changes of states *C* (red) and *M* (black) and Ca^2+^-bound Tn (gold) under standard conditions (Table 1). Adjustments in standard conditions (Table 2) alter the decay rate of Ca^2+^-bound Tn (Δ*x*_CaB_).

In the overlap preparation, the structural changes related to meridional 1/38.5 nm^−1^ reflection intensity are more complex (Matsuo et al., 2010), showing intensity changes with positive and negative slopes over the time course of a twitch (Figure 6). The early rise in reflection intensity correlates with a brief period at the beginning of the Ca^2+^ pulse in which the model predicts a rise in Ca^2+^-bound Tn and state *C* of Tm before the transition to state *M* of Tm begins. The large negative change in reflection intensity has roughly the same time course as the calculated fraction of Tm in the *M* position (Figure 6). The minimum reflection intensity comes at a time after the Ca^2+^ transient has decayed and the calculated *M* state has peaked.

The calculated decay rate of Ca^2+^-bound Tn can be made more rapid than the measured decay of fluo-3 fluorescence by increasing rate constants, *K*_1_ *k*_−1_, at fixed *K*_1_ (Figure 6, Table 2). However, both absolute rates and the competition between states *M* and *B* for Tm in state *C* (Figure 1) must be made more extreme to hold constant the calculated tension transient (Figure 3). Thus, a balance of competing factors explains the correlation between the calculated Ca^2+^-bound state of Tn and the fluo-3 fluorescence transient in the overlap preparation (Figure 6).

### Predicted time course of crossbridge-dependent Ca^2+^-bound Tn

**Figure 7:**
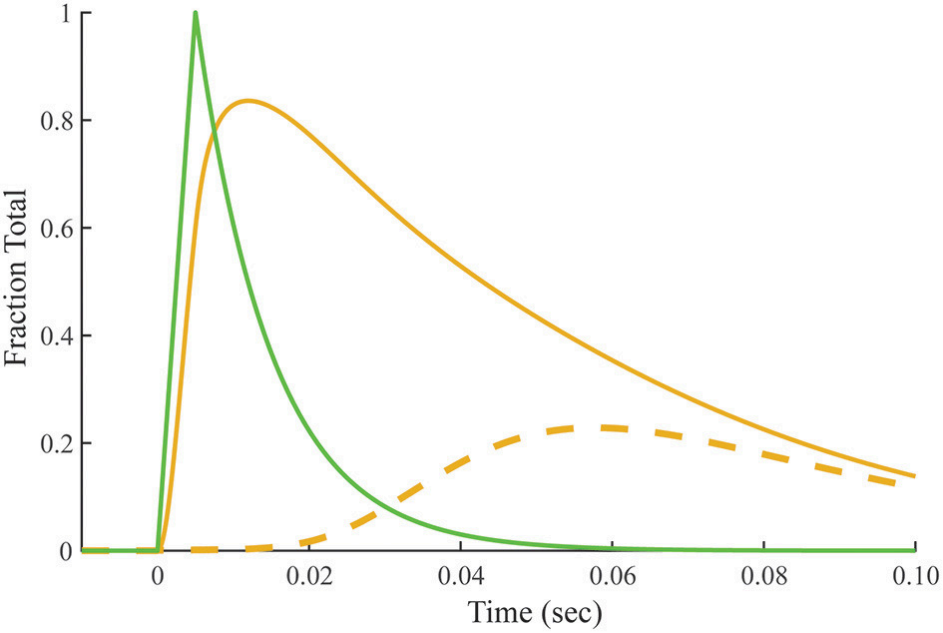
**Predicted time course of crossbridge-dependent Ca^2+^-bound Tn**. Crossbridge-dependent Ca^2+^-bound Tn is the residual (dashed line) obtained by subtracting Ca^2+^-bound Tn calculated for the non-overlap preparation (Figure 4) from Ca^2+^-bound Tn calculated for the overlap preparation (solid gold line; Figure 3). The stimulus for both conditions (green) is taken from Figure 2.

Crossbridge-dependent Ca^2+^-bound Tn is the difference between Ca^2+^-bound Tn calculated for overlap and non-overlap conditions, holding the transient of free Ca^2+^ constant (Figure 7). The residue is a pool of crossbridge-dependent Ca^2+^-bound Tn, which represents about 30% of the total area under the curve. The rise in the pool of crossbridge-dependent Ca^2+^-bound Tn begins near the end of the free Ca^2+^ transient and continues during the decay of the tension transient. Peaking at ~60 ms, the time course of rising crossbridge-dependent Ca^2+^-bound Tn correlates most closely with the time course of the declining phase in meridional 1/38.5 nm^−1^ reflection intensity, which reaches a minimum at ~70 ms.

## Discussion

A comprehensive model of thin filament regulation presented here supports the hypothesis that Ca^2+^-bound Tn has a causal relationship with the structure of Tn in the thin filament and not with the pool of Ca^2+^ represented by the intensity of fluo-3 fluorescence. Rather than recapitulating the decay of the fluo-3 fluorescence change in the non-overlap preparation of frog muscle, the model presented here generates transients for Ca^2+^-bound Tn and position *C* of Tm-Tn that match transient changes in intensity of the meridional 1/38.5 nm^−1^ reflection in response to Ca^2+^ stimulus. The model predicts similar rates of Ca^2+^ dissociation from the regulatory sites of Tn in the first 50 ms following stimulation of both preparations, overlap and non-overlap. After this period, the model predicts that decays of Ca^2+^-bound Tn in the two preparations diverge, hence demonstrating a fraction of Ca^2+^-bound Tn that persists owing to crossbridge interaction. Given the same free Ca^2+^ transient for both overlap and nonoverlap conditions, calculations of Ca^2+^-bound Tn, and crossbridge-dependent Ca^2+^-bound Tn correlate with the time courses of positive and negative changes in intensity of the meridional 1/38.5 nm^−1^ reflection, respectively, suggestive of a causal relationship. By reproducing the twitch of a well-studied physiologic system given a highly reproducible experimental Ca^2+^ transient, we achieve a proof of concept for the model presented here.

Fluo-3 may be responding to an exchangeable pool of Ca^2+^ bound to Ca^2+^ buffers in the sarcoplasm (Cannell and Allen, 1984; Baylor and Hollingworth, 1998). Small diffusible Ca^2+^ binding molecules such as ATP and parvalbumin in the myofilaments can facilitate the diffusion of Ca^2+^ (Feher, 1984) and thereby possibly reduce random non-uniform reactivation events in relaxing myofibrils. However, a fixed Ca^2+^ buffer also prolongs elevated Ca^2+^ in the cytosol at all sarcomere lengths, regardless of the status of Ca^2+^-bound Tn. The decay of the fluo-3 fluorescence may represent a compromise based on the Ca^2+^ requirements of the working and relaxed muscle.

We assume that the Ca^2+^ sequestration apparatus has the capacity to prevent a significant rise in free Ca^2+^ resulting from a pool of crossbridge-dependent Ca^2+^-bound Tn during a twitch. Aside from equilibrium binding of Ca^2+^ to buffering agents represented by the fluo-3 fluorescence intensity, Ca^2+^ is actively transported from the sarcoplasm by the Ca^2+^ pump of the sarcoplasmic reticulum (SR Ca^2+^-ATPase). Previous results from amphibian fast skeletal muscle using inhibitors of SR Ca^2+^-ATPase have shown that the decay and not the peak in free Ca^2+^ depends on active Ca^2+^ transport (Jiang et al., 1996; Westerblad and Allen, 1996; Même et al., 1998; Caputo et al., 1999). The delay in onset argues against a significant contribution to the rise and decay process of free Ca^2+^ sparks by Ca^2+^ dissociating from the pool of crossbridge-dependent Ca^2+^-bound Tn. We suggest the rate and load of Ca^2+^ dissociating from crossbridge-dependent Ca^2+^-bound Tn is within the capacity of Ca^2+^ buffers and SR Ca^2+^-ATPase to move into the SR without effecting a significant increase in sarcoplasmic free Ca^2+^.

One assumption of the model is that cooperative activation depends exclusively on cycling crossbridges. Although, Equation (1) has the distinction of being a more general form of the Hill equation (Zot et al., 2012), the resulting system of ODE we derive here are mathematically compatible with any logistic function. Hence, the model presented here is limited to a mechanism of regulation in which a steady-state process fully accounts for cooperative transitions between *C* and *M* states.

A second assumption of the model is that activated state, *M*, is proportional to the fraction of maximum tension. This simplification is consistent with the proposal that tension bearing crossbridges are excluded from thin filament states *C* and *B* (Lehman et al., 2000).

A third assumption of the model is that Ca^2+^ binding to the regulatory sites of TnC is uncoupled from the process of activation when the complex of Tm-Tn is in positions *C* and *M*. Consequently, we model the *C* position as favored at equilibrium. Tm occupies either *C* or *B* positions in reconstructions of skeletal and cardiac filaments, respectively, but the complex of Tm-Tn is positioned exclusively in *C* with Ca^2+^ present (Lehman et al., 2000). There is general agreement that Ca^2+^ is required to release Tm-Tn from the *B* position. A possible, albeit more complicated, mechanism is for Ca^2+^ to have a second, independent action, namely, to perturb the equilibrium position of the Tm-Tn complex. This latter possibility is difficult to reconcile with an uncoupling mechanism.

Although, we do not address a specific myopathy, we suggest that results presented here can be extrapolated to mechanisms underlying disease. By employing a model that accounts for the Ca^2+^ regulatory properties of Tn, we provide a satisfying explanation for the events of contraction arising from a transient of free Ca^2+^. The model presented here is consistent with previous experimental results showing non-cooperative Ca^2+^ binding to regulated actin in the presence or absence of rigor crossbridges and recapitulates the complex cooperative relationship between Ca^2+^-binding and force in the steady-state (Figure 2). Of the eight adjustable parameters (Table 2), we have consistently published results in which *K*_1_ alone is freely adjusted and *K*_3_ and *K*_5_ vary in a prescribed manner. A recent study shows that the model presented here can inform experiments that explain how a mutation in TnC alters the Ca^2+^ sensitivity of cardiac myofilaments associated with the hypertrophic state of the heart (Zot et al., 2016b). A previous study shows that the model presented here can fully explain the depressing effect of Ca^2+^-insensitive mutant TnC on cooperative activation of skeletal muscle fibers (Zot et al., 2009). Hence, a growing body of experimental results in cardiac and skeletal muscle, mutant and wild type preparations, reconstituted and intact systems, and steady-state and transient conditions are explained by the same model and highly constrained parameters of this model. As a robust and reliable predictor of transient and steady-state changes in thin filament structure related to Ca^2+^-bound Tn, the model presented here is capable of guiding future experiments to uncover mechanisms by which mutations in excitation-contraction coupling lead to pathological conditions.

## Author contributions

Each of the authors contributed significantly to the conception and design of the work, drafting and revision of the manuscript, and final approval of the version to be published. Both authors agree to be accountable for all aspects of the work.

### Conflict of interest statement

The authors declare that the research was conducted in the absence of any commercial or financial relationships that could be construed as a potential conflict of interest.

## Abbreviations

Troponin: Tn
tropomyosin: Tm
*B*: state of Tm in the blocking position
*C*: state of Tm in the central position
*M*: state of Tm in the myosin-dependent position.
