# Supplementary material for: Modeling Ca2+-Bound Troponin in Excitation Contraction Coupling
Source: Front Physiol. 2016 Sep 21;7:406. doi: 10.3389/fphys.2016.00406 (PMC5030304; doi:10.3389/fphys.2016.00406)
Supplement: Supplementary file 1 [file DataSheet1.PDF]

Supplemental Materials

Matlab Program

```
%fast_twitch_Transient_with_exp_pulses.m, by J. E. Hasbun (3/2016)
%made from the original fast_twitch_Transient.m of 11/2009.
%On the transient state equations for fast twitch muscle
%The capability to include pulses is incorporated as well
%In collaboration with H. Zot and Minh v Guyen
%
function fast_twitch_Transient_v1
global k0 k_0 k1 k_1 k2 k_2 k3 k_3 k4 k_4 k5 k_5 alpha n
global ip p1 beta tp ttp delta
k0=50; %K0k_0
k_0=50;
k1=80000; %K1k_1
k_1=100;
k2=25; %K2k_2
k_2=15;
k3=8000; %K3k_3
k_3=100;
k4=25; %K4k_4
k_4=150;
k5=800; %K5k_5
k_5=100;
alpha=3.25;
n=5;
ttp=.005; %time to peak of the pulse
delta=18;  %peak height in uM
% user enter rate (beta) and time to pulse (tp)
% adjust tp as needed to stablize calculation before pulse
p1(1)=1; beta(1)=100; tp(1)=0.055;
%
%time range and step size
t0=0.0; tmax=0.15; tstep=0.0001;
%store constants in workspace
inpName={'k0';'k_0';'k1';'k_1';'k2';'k_2';'k3';'k_3';....

'k4';'k_4';'k5';'k_5';'alpha';'n';'ttp';'delta';'t0';'tmax';'tstep'};
inpValue=[k0;k_0;k1;k_1;k2;k_2;k3;k_3;....
    k4;k_4;k5;k_5;alpha;n;ttp;delta;t0;tmax;tstep];
input_T=table(inpName,inpValue);
assignin('base','input_T',input_T);
%
%initial values
M0=0.0; B10=0.9; B20=0.0; B30=0.0; T20=0.0; T30=0.0;
ic1=[M0;B10;B20;B30;T20;T30];
opt=odeset('AbsTol',1.e-4,'RelTol',1.e-4);%user set Tolerances
[t,w]=ode45(@FT_Transient_der,(t0:tstep:tmax),ic1,opt);
M=w(:,1); B1=w(:,2); B2=w(:,3); B3=w(:,4); T2=w(:,5); T3=w(:,6);
T1=1.-B1-B2-B3-T2-T3; C=1.-M-B1-B2-B3; CaB=B2+B3+T2+T3;
```

```
B=B1+B2+B3; T=T1+T2+T3;
Bb=B2+B3; Tt=T2+T3;
%transient Ca
caConc=exp_pulse(t)*delta;
%store calculation in workspace
runout_T=table(t,M,C,B,B1,B2,B3,T1,T2,T3,CaB,caConc,T,Bb,Tt);
assignin('base','runout_T',runout_T);
%plot calculation
figure
hold on
plot(t,exp_pulse(t),'y')   %look at the pulse use
plot(t,M,'k')
plot(t,B,'c')
plot(t,Bb,'b')
plot(t,Tt,'m')
plot(t,C,'r')
plot(t,B2+B3+T2+T3,'g')
hold off
legend('Pulse','M','B','Bb','Tt','C','Bb+Tt')
% to save workspace uncomment below
% writetable (fluo3Data_T,'fluo3Data_T.xlsx');
% writetable (input_T,'input_T..xlsx');
% writetable (runout_T,'runout_T.xlsx');
%

function derivs = FT_Transient_der(t,w)
%The transient state derivatives for fast twitch muscle
global k0 k_0 k1 k_1 k2 k_2 k3 k_3 k4 k_4 k5 k_5 alpha n delta
M=w(1); B1=w(2); B2=w(3); B3=w(4); T2=w(5); T3=w(6);
ft=exp_pulse(t);
T1=1.-B1-B2-B3-T2-T3;
C=1.-M-B1-B2-B3;
F0=k0*C.*(1.+M*(alpha-1)).^n-k_0*M;
F1=k1*C*T1+k_4*B2-(k_1+k4*delta*ft)*B1;
F2=k4*delta*ft*B1+k3*T2.*C+k_4*B3-(k_4+k_3+k4*delta*ft)*B2;
F3=k4*delta*ft*B2+k5*T3.*C-(k_4+k_5)*B3;
F4=k2*delta*ft*T1+k_3*B2+k_2*T3-(k_2+k2*delta*ft+k3*C).*T2;
F5=k2*delta*ft*T2+k_5*B3-(k_2+k5*C).*T3;
derivs=[F0;F1;F2;F3;F4;F5];

function [y]=exp_pulse(t)
%This function creates the pulse type
global ip p1 beta tp ttp
if(ip==0)          %constant pulse case
    y=p1(1);
else               %ip pulses
    y=0;
    for i=1:ip
        tUp=t-(tp(i)-ttp); tDn=t-tp(i);
        [maskUp,maskDn]=theta(tUp,tDn);
        upSide=maskUp.*fun_line(t-(tp(i)-ttp),1/ttp,0);
        dnSide=maskDn.*exp(-beta(i)*(t-tp(i)));
```

```
        y=y+p1(i)*(upSide+dnSide);
    end
end

function [yUp,yDn]=theta(tUp,tDn)
%step function; i.e., y=0 if t <0, y=0.5 if t=0, and y=1 if t >0
%Reference https://www.physicsforums.com/threads/creating-a-unit-step-
function-in-matlab.288638/
yUp=tUp./abs(tUp); %this performs the same operation as the matlab
"sign"
yDn=tDn./abs(tDn);
yUp=yUp-yDn;
yUp(isnan(yUp))=2.0;
yUp=0.5*yUp;
yDn(isnan(yDn))=-1.0;
yDn=0.5*(yDn+1);

function y=fun_line(x,m,b)
y=m*x+b;
```

Supplemental Figures and Tables

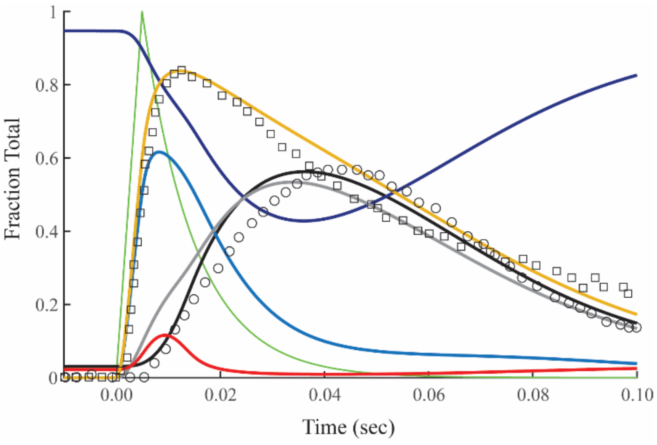

Figure S1. Example of near fit of tension data. Values used for calculations are inTable S1. Time courses of principle states of the thin filaments with overlap. Plotted as a function of a pulse of free calcium (green) are calculated transients of states *B* (dark blue), *C* (red), and *M* (black). Total Ca^{2+}-bound Tn ($B_2 + B_3 + T_2 + T_3$; gold) is broken down into fast and slow components based on Ca^{2+}-bound *B* states ($B_2 + B_3$; blue) and Ca^{2+}-bound *T* states ($T_2 + T_3$; gray), respectively. On the same scale as calcium-bound troponin are measured tension (circles) and fluo-3 fluorescence (squares) transients of muscle fibers stretched to resting length and stimulated by a single impulse (reproduced from Matsuo et al., 2010).

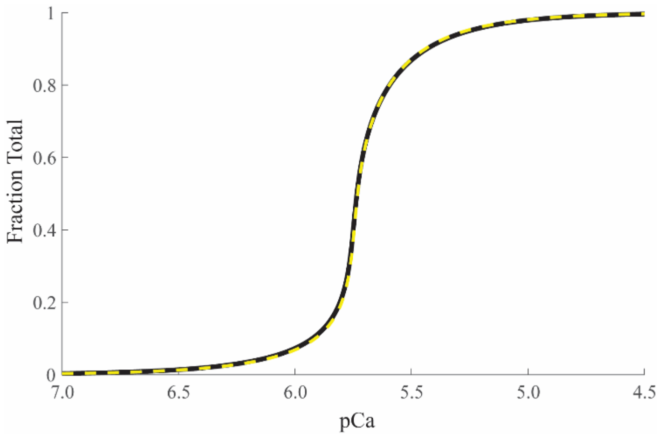

Figure S2. Alignment of steady-state calculations for faster k_{-4}. The off rate of Ca^{2+} from $B2$ and $B3$, k_{-4}, is 150 s^{-1} for standard conditions (dark blue) and is increased 1.33 fold to 199.5 s^{-1} (dashed yellow). A faster k_{-4} decreases K_4 and increases K_2/K_4. Because $K_2/K_4 = K_1/K_3 = K_3/K_5$, K_1 is the only free parameter. K_1 is increased 1.1 fold to achieve the alignment.

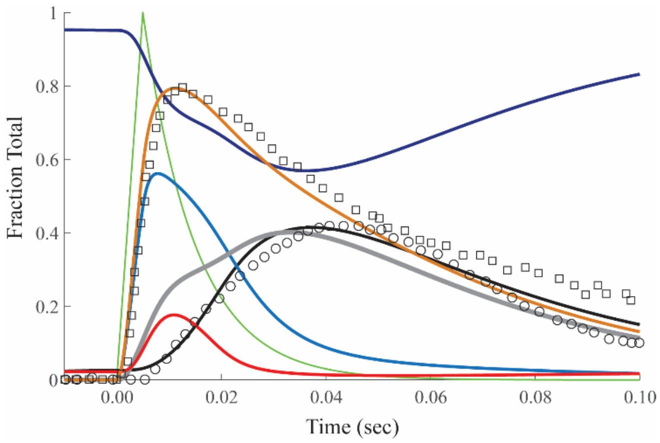

Figure S3. Calculated transients using faster k_{-4} for overlap preparation. Plotted as a function of a pulse of free calcium (green) are calculated transients of states B (dark blue), C (red), and M (black). Total Ca^{2+}-bound Tn ($B_2 + B_3 + T_2 + T_3$; gold) is broken down into fast and slow components based on Ca^{2+}-bound B states ($B_2 + B_3$; blue) and Ca^{2+}-bound T states ($T_2 + T_3$; gray), respectively. On the same scale as calcium-bound troponin are measured tension (circles) and fluo-3 fluorescence (squares) transients of muscle fibers stretched to resting length and stimulated by a single impulse (reproduced from Matsuo et al., 2010). Constants for the calculation are in Table S1.

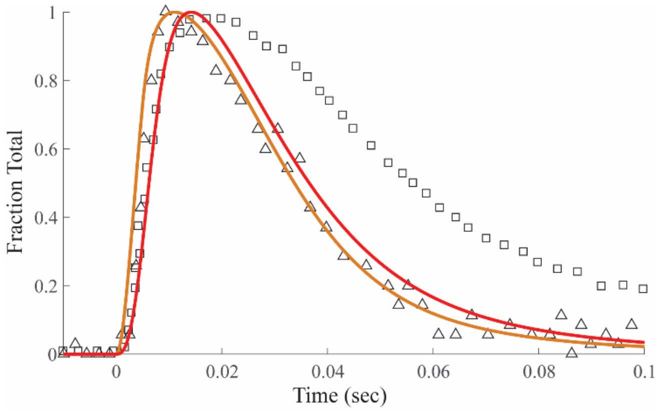

Figure S4. Compare faster off rate with meridional 1/38.5 nm^{-1} reflection intensity in non-overlap preparation. The off rate of Ca^{2+}, k_{-4}, is increased to 199.5 s^{-1} compared with standard conditions. Constants used in calculation are in Table S1. Plotted on the same scale are the calculated temporal change of state *C* (red) and calcium bound to troponin (gold) under standard conditions (Table 1) with k'_0 set to null.

Table S1
Constants Used for Calculations in
Supplementary Figures

Constant	Fig S1	Fig. S3	Fig. S4
K'_0	1	1	0
K'_0k_{-0}	100	40	0
k_{-0}	100	40	0
K_1	800	880	880
K_1k_{-1}	80000	132000	132000
k_{-1}	100	150	150
K_2	1.67	1.67	1.67
K_2k_{-2}	25	25	25
k_{-2}	15	15	15
K_3	80	66.165	66.165
K_3k_{-3}	8000	9924.812	9924.812
k_{-3}	100	150	150
K_4	0.167	0.125	0.125
K_4k_{-4}	25	25	25
k_{-4}	150	199.5	199.5
K_5	8	4.975	4.975
K_5k_{-5}	800	746.226	746.226
k_{-5}	100	150	150
α	3.25	3.25	3.25
n	5	5	5

Table S2
Equilibrium Constants used
Figure S3

	Standard	Fast k_{-4}
K'_0	1	1
K_1	800	880
K_2	1.67	1.67
K_4	0.167	0.1256
α	3.25	3.25
n	5	5
